# Child Welfare System Involvement Among Children With Medical Complexity

**DOI:** 10.1177/10775595211029713

**Published:** 2021-07-05

**Authors:** Corry Azzopardi, Eyal Cohen, Karine Pépin, Kathy Netten, Catherine Birken, Sheri Madigan

**Affiliations:** 1 Suspected Child Abuse and Neglect Program, Division of Paediatric Medicine, The Hospital for Sick Children, Toronto, Ontario, Canada; 2 Child Health Evaluative Sciences, The Hospital for Sick Children, Department of Paediatrics and Institute of Health Policy, Management & Evaluation, Edwin S.H. Leong Centre for Healthy Children, University of Toronto, Ontario, Canada; 3 Department of Paediatric, Centre Hospitalier Universitaire Sainte-Justine, Université de Montréal, Quebec, Canada; 4 Department of Social Work, The Hospital for Sick Children, Toronto, Ontario, Canada; 5 Department of Paediatrics, University of Toronto, Child Health Evaluative Sciences, SickKids Research Institute, Toronto, Ontario, Canada; 6 Department of Psychology, University of Calgary, Alberta Children's Hospital Research Institute, Calgary, Alberta, Canada

**Keywords:** children with medical complexity, child maltreatment; child welfare, child protection, maltreatment risk, pediatric health

## Abstract

Children with medical complexity may be at elevated risk of experiencing child maltreatment and child welfare system involvement, though empirical data are limited. This study examined the extent of child welfare system involvement among children with medical complexity and investigated associated health and social factors. A retrospective chart review of children with medical complexity (N = 208) followed at a pediatric hospital-based complex care program in Canada was conducted. Descriptive statistics and odds ratios using logistic regression were computed. Results showed that nearly one-quarter (23.6%) had documented contact with the child welfare system, most commonly for neglect; of those, more than one-third (38.8%) were placed in care. Caregiver reported history of mental health problems (aOR = 3.19, 95%CI = 1.55–6.56), chronic medical conditions (aOR = 2.86, 95%CI = 1.09–7.47), and interpersonal violence or trauma (aOR = 17.58, 95%CI = 5.43–56.98) were associated with increased likelihood of child welfare system involvement, while caregiver married/common-law relationship status (aOR = 0.35, 95%CI = 0.16–0.74) and higher number of medical technology supports (aOR = 0.75, 95%CI = 0.57–0.99) were associated with decreased likelihood. Implications for intervention and prevention of maltreatment in children with high healthcare needs are discussed.

Children with special healthcare needs may be uniquely vulnerable to experiencing adversities in childhood, including child maltreatment and child welfare system involvement. Among children with chronic conditions, specific subsets of children such as those with medical complexity, may be at particularly high risk. Representing less than one percent of the general pediatric population, yet accounting for one-third of all pediatric healthcare spending in North America, children with medical complexity are broadly defined as those with chronic medical conditions requiring specialized care, functional disability, high healthcare system utilization, and extensive family-identified needs (Cohen et al., 2011; Dewan & Cohen, 2013). Substantial advances in medical technology and treatment efficacy have resulted in improved survival rates, increased life span, and more time spent in family home versus hospital environments (Hatzmann et al., 2008). The enduring and arduous care demands placed upon parents and other significant caregivers can, at times, exceed their abilities and resources, potentially placing children with medical complexity at risk of harm. Concerns of child abuse or neglect, or daily childcare needs that outweigh the financial, physical, or emotional capacities of caregivers, may result in referral to child protection services. In some circumstances, temporary or permanent placement in an out-of-home care setting may occur, either voluntarily or involuntarily (Friedman et al., 2016). Despite being a highly impactful problem, little is known about the nature and scope of child welfare system involvement in the lives of children with medical complexity and their families. The goals of the current study were therefore to examine the extent and predictors of child welfare system involvement in this vulnerable population.

## Health and Service Needs of Children With Medical Complexity

Children with medical complexity typically require care from numerous service providers across multiple disciplines and sites to address their wide-ranging physical, neurological, developmental, psychological, and social needs (Cohen, Berry, et al., 2012). Some require intensive daily therapies, complicated pharmaceutical and nutritional regimens, and dependence on invasive medical technologies, such as tracheostomy or gastrostomy tubes, to accomplish the basic tasks of daily living and promote development. They may need recurrent and prolonged hospitalizations, frequent outpatient visits with primary care providers and specialists, and in-home care by nursing and other allied healthcare providers. The quality of clinical care, sometimes marked by poor infrastructure, service fragmentation, communication breakdowns, and navigation challenges, can increase the burden relegated to families and place children at higher risk for adverse outcomes (Allshouse et al., 2018; Cohen, Lacombe-Duncan, et al., 2012; Dewan & Cohen, 2013).

Much of the responsibility for management of comprehensive care in the home falls upon the families of children with medical complexity. Parents and other significant caregivers report spending up to 20 hours per week providing direct homecare, plus a median of 2 hours per week coordinating services (Kuo et al., 2011). The extent of attention, supervision, and routine required can be a tremendous physical, psychological, and economic burden for families, particularly those without sufficient support networks and respite resources. While some parents find a meaningful sense of purpose in life and show profound resilience, others report feeling overwhelmed and exhausted by the acute and chronic caregiving pressures thrust upon them and subsequently endure poor health-related quality of life (Hatzmann et al., 2008). Caring for a child with special healthcare needs has been associated with elevated stress, physical and mental health problems, grief and trauma, sleep deprivation, employment loss, financial hardship, and social isolation (Cousino & Hazen, 2013; Kuo et al., 2011; Mackay et al., 2019; Miodrag et al., 2015; Smith et al., 2013). A recent systematic review and meta-analysis of 26 studies found that caregivers, specifically mothers, of children with chronic illness experience clinical depression and anxiety at significantly higher rates than those with healthy children (Cohn et al., 2020). The breadth of caregiver adversities, in the context of intersecting structural and systemic sources of marginalization, inequity, and stigma, has the potential to cumulatively impact child safety and well-being.

## Maltreatment Risk in Children With Medical Conditions

Child maltreatment refers to the harm, or risk of harm, to a child in the form of neglect, physical abuse, sexual abuse, and/or emotional abuse, including exposure to violence in the home. Child maltreatment can have pervasive and enduring effects on neurobiological development and physical and psychological functioning over the lifespan (Afifi et al., 2014; Anda et al., 2006). Rates of abuse and neglect among children with medical complexity, and their involvement with the child welfare system, are difficult to discern due to challenges in surveillance and discrepancies in operational definitions and classification methods. Extant maltreatment research commonly uses medical terms such as “disability” or “special needs” relatively broadly, conflating discreet categories of physical and mental health diagnoses, disregarding severity of illness and impairment, failing to differentiate between pre-existing disabilities and those resulting from maltreatment, and neglecting to account for the influence of overlapping risk factors (Kendall-Tackett et al., 2005; Leeb et al., 2012). Moreover, most available child welfare incidence data lack universal language for designating children with medical complexity within the child welfare and medical foster care systems (Williams et al., 2017). Consequently, empirical data explicating the scope of the problem are limited.

Notwithstanding research shortcomings, young children with a range of chronic illnesses and disabilities, compared to their healthy counterparts, have been found to be at increased risk of maltreatment and over-represented in the child welfare system (Jaudes & Mackey-Bilaver, 2008; Jones et al., 2012; Stein et al., 2013; Turner et al., 2011). In their population-based epidemiological study of 50,278 school children, Sullivan and Knutson (2000) reported a 31% prevalence rate of maltreatment among children with diagnosed disabilities, compared to 9% for those without disabilities. The children with disabilities in this sample were also more likely to endure multiple forms (e.g., both neglect and physical abuse) and recurring episodes of maltreatment. While little data exist on child protection referrals and investigation outcomes for children with medical complexity specifically, evidence of increased risk can be drawn from studies examining other known chronic health conditions. For example, children with HIV (Azzopardi et al., 2014) and children with asthma (Hellyer et al., 2013) have been shown to be reported to child protection services and placed in care at higher rates than the general pediatric population. Highlighting nuances in research findings, type of childhood victimization and level of risk may vary across diverse forms of disabilities (Turner et al., 2011). Maclean et al. (2017) and Van Horne et al. (2015) found increased risk of substantiated maltreatment involving children with certain diagnoses (e.g., conduct disorders, spina bifida) but not others (e.g., Down syndrome). This may be related to the particular characteristics of the child’s condition (e.g., hyperactivity) or caregiver (e.g., maternal age, mental health) and associated degree of risk.

In the Canadian province of Ontario, the site of the current study, an estimated 37% of all substantiated child maltreatment investigations in 2018 involved a child with at least one known functioning concern that reflects physical, emotional, cognitive, or behavioral problems (Fallon et al., 2020). Of those identified with a physical disability, intellectual/developmental disability, or failure to meet developmental milestones, 32% were placed in care following the initial child protection investigation, a rate significantly higher than those without a known functioning concern (Joh-Carnella & Allan, 2020). Data from the United States suggest that up to half of the children investigated by child protection services and placed in care have chronic physical or mental health conditions and special needs (Mekonnen et al., 2009; Ringeisen et al., 2008; Stein et al., 2013), with approximately 10% of those in care identified as medically fragile or complex (American Academy of Pediatrics, n.d.).

The extent of child maltreatment and child welfare system involvement among children with complicated medical profiles has precipitated the study of interconnected variables associated with greater risk. Theoretical and empirical literature suggests that some medically fragile children are more vulnerable to mistreatment due to societal stigma undermining their perceived worth, their extensive care needs and dependency on caregivers, cognitive deficits that impede their understanding of abuse, communication impairments that prevent their disclosure of abuse, and excessive parenting stress and frustration, particularly if the medical condition involves behavioral manifestations (Brodie et al., 2017; Fisher et al., 2008; Hibbard et al., 2007; Maclean et al., 2017). Level of risk linked to child age and sex varies. In Sullivan and Knutson’s (2000) study, younger age appeared to increase risk of maltreatment in some disabilities but not others; and boys with disabilities were more likely to be maltreated than girls with disabilities, though possibly a function of higher prevalence of disability status among males. Likelihood of child abuse and neglect is generally compounded by interacting familial and environmental risk factors such as caregiver substance use, physical and mental illness, intimate partner violence, parenting stress, and limited supports and resources (Mulder et al., 2018; Stith et al., 2009); however, it is less clear how these predisposing variables uniquely impact children with medical complexity.

In addition to the heightened risk of maltreatment, this subpopulation of medically complex children may be more likely to come to the attention of the child welfare system due to their high visibility in the community and frequent contact with mandated reporters involved in their care. Moreover, the stress of caregiving, and thus the need for child protection services, may be exacerbated by deficiencies in the pediatric healthcare and social services sectors. To illustrate this point, some parents have been known to resort to entering into temporary care agreements with the child welfare system for the purpose of obtaining necessary daily care for their children with special healthcare needs, at times resulting in permanent custody loss (Ombudsman Ontario, 2005). Child health and developmental outcomes may be further compromised by placement instability and unmet medical needs in out-of-home care settings (Szilagyi et al., 2015; Weil et al., 2018).

## The Current Study

To advance our understanding of the nature and scope of child welfare system involvement among children with medical complexity, the objective of the current study was twofold. (1) To examine the extent of child welfare system involvement among a sample of children with medical complexity followed by a structured complex care program situated in a tertiary pediatric hospital. (2) To investigate a range of child and caregiver health and social factors associated with the likelihood of child welfare system involvement among children with medical complexity. Informed by our clinical experiences and existing literature, we hypothesized increased odds of involvement with the child welfare system in families with financial stress and single caregiver relationship status; in children with more health diagnoses, hospitalizations, days in hospital, technology assistance, developmental delay, and behavioral problems; and in caregivers with mental health problems, medical conditions, and interpersonal violence or trauma.

## Method

### Design and Participants

This study was a collaboration between the Suspected Child Abuse and Neglect Program and the Complex Care Program at the Hospital for Sick Children (SickKids), a large academic health sciences center in Toronto, Ontario, Canada. These tertiary pediatric programs provide integrated and specialized interprofessional care and consultation in the distinct areas of child maltreatment and complex medical conditions. The Complex Care Program, from which the study sample was recruited, was established in 2006 to provide care coordination and supports to children with medical complexity followed at SickKids (Cohen, Friedman, et al., 2012; Cohen, Lacombe-Duncan, et al., 2012). Its multidisciplinary team is comprised of pediatricians, nurse practitioners, social workers, dietitians, and information coordinators. The program also has a number of community-based satellite clinics within the catchment area of SickKids. Referrals to the program can be initiated by any healthcare provider. The children referred have a diverse range of diagnoses; most are multi-system. Some have suspected genetic conditions without an overarching diagnosis. Common to many is neurological impairment, which arises from a heterogenous group of static and progressive diseases that affect the central and peripheral nervous systems. Examples of diagnostic categories include cerebral palsy, spina bifida, and trisomy 18. While difficult to quantify with empirical certainty, we estimate based on our anecdotal clinical observations that for less than 2% of our patient population, etiology of medical conditions was related to an event that would have interfaced with child protection (e.g., abusive head trauma resulting in severe chronic physical and cognitive impairment).

Children followed by the Complex Care Program may intersect with the child welfare system before or during their involvement with our program when concerns about their best interests, protection, and well-being have been identified. The child welfare system in Ontario is currently comprised of 50 Children’s Aid Societies and Indigenous Child and Family Well-Being Agencies mandated to protect children from harm, and risk of harm, and to support families, as per the Child, Youth and Family Services Act (CYFSA, 2017; Ontario Association of Children’s Aid Societies, 2020).

Following institutional research ethics board approval, a retrospective chart review was conducted between 2015 and 2016, in adherence with recommended guidelines (Gearing et al., 2006; Vassar & Holzmann, 2013). The health records of all patients (N = 208) followed by the Complex Care Program at the time of data collection were included in the review. All were under the age of 18-years at the time of referral to the program and met three or more of the following conditions for medical complexity: ≥1 chronic health condition affecting ≥2 organ systems; ≥2 chronic prescription medication(s), special diet requirement, or medical technology dependence; healthcare delivered by a licensed practitioner in ≥3 settings; ≥2 medical subspecialists involved; ≥2 allied health professionals involved. These conditions constitute the program eligibility criteria. They were derived from research and adapted to the specific context of Ontario based on guidance from the Provincial Council of Maternal and Child Health for a standard operating definition for children with medical complexity (Cohen, Lacombe-Duncan, et al., 2012; Complex Care Kids Ontario, 2017).

### Data Collection and Study Variables

Informed by the study protocol for systematic data abstraction, a physician and trained research assistants used a structured data collection tool developed for the purpose of this review to extract de-identified sociodemographic, medical, and psychosocial data from the electronic health records of patients meeting study inclusion criteria. All were double-coded by two independent reviewers for accuracy. Rate of consensus was high (>90%) and discrepancies were resolved through conferencing. Where pertinent psychosocial data were missing from the charts or documented in a manner that required clarification, the responsible clinician was queried. This occurred in less than 5% of the cases.

*Child welfare characteristics* consisted of known past or present child welfare system involvement including documented contact prior to and/or while being followed by the Complex Care Program, reason for child welfare referral (neglect, intimate partner violence, physical abuse, emotional abuse, sexual abuse, other including caregiver capacity concerns [e.g., due to mental health, substance use] and voluntary involvement with no identified protection concern, or unknown), and known past or present child welfare placement in temporary or permanent out-of-home care following investigation, based on collateral report or caregiver self-report.

*Sociodemographic variables* consisted of the child’s age (in months) at the time of referral to the Complex Care Program, child’s sex at birth (male or female), primary caregiver relationship status (married/common-law or single), annual family income (low = <$40,000 [based on the regional low income cut-off rate for an average family of four] or medium/high > $40,000), and financial assistance from government or charity for health-related expenses not covered by the universal provincial health insurance plan (received or not received).

*Child clinical variables* consisted of the total number of physical and mental health diagnoses, number of hospitalizations at the current hospital since birth, number of days hospitalized as an inpatient since birth, number of prescription medications, number of medical technology supports (e.g., tracheostomy tube for breathing, gastrostomy tube for feeding), presence of diagnosed developmental delay or behavioral problem, and palliative care status (palliative or not palliative).

*Caregiver clinical variables* consisted of the known presence of a caregiver history of chronic medical condition or physical limitation, mental health problem, and interpersonal violence or trauma (e.g., childhood abuse, intimate partner violence), based on collateral report or caregiver self-report.

All of the above data pertaining to the child, including child welfare and caregiver characteristics, were documented in designated sections of the child’s health record. Child welfare system involvement and caregiver variables were coded according to their *known* presence based on chart recordings (0 = yes; 1 = no/unknown).

### Data Analysis

Data were analyzed using SPSS 24. Descriptive statistics were calculated, including the count (percentage) for categorical variables, as well as the mean (M), standard deviation (SD), and range for continuous variables. To examine the association between sociodemographic, child, and caregiver characteristics and past or present child welfare system involvement, odds ratios (ORs) were computed. Using logistic regression, unadjusted ORs and adjusted ORs (aORs) were computed, as well as 95% confidence intervals (CIs), to assess the odds of different sociodemographic, child, and caregiver characteristics predicting past or present child welfare system involvement. Adjusted models included two covariates (child age and sex). Analyses were run on all participants for whom there was data available regarding child welfare system involvement (N = 208). A power analysis demonstrated that a sample size of 121 participants was required in order to detect a small to medium effect size in regression analyses, using a power value of .80 and a probability level of .05. Thus, our sample size of 208 was adequate.

## Results

Table 1 presents sociodemographic and clinical characteristics of the sample of children with medical complexity, without and with child welfare system involvement.

**Table 1.** Sociodemographic and Clinical Characteristics of Children With Medical Complexity, Without and With Child Welfare System Involvement.

	Without Child Welfare Involvement (N = 159)		With Child Welfare Involvement (N = 49)		Total Sample (N = 208)	
	M (SD)	N (%)	M (SD)	N (%)	M (SD)	N (%)
Sociodemographic Variables						
Child age (in months)	93.49 (64.29)		102.88 (56.55)		99.53 (62.44)	
Child sex (male)		83 (52.2)		20 (40.8)		103 (49.5)
Caregiver relationship status (married/common law)		121 (76.1)		25 (51.0)		146 (70.2)
Family income (low)		58 (36.5)		28 (57.1)		86 (41.3)
Financial assistance (received)		117 (73.6)		38 (77.6)		155 (74.5)
Child Clinical Variables						
Number of health diagnoses	13.64 (4.93)		14.37 (4.56)		13.81 (4.84)	
Number of hospitalizations	12.89 (12.43)		12.63 (10.36)		12.83 (11.95)	
Number of days in hospital	169.15 (172.69)		164.27 (132.36)		168.00 (163.80)	
Number of medications	9.64 (6.45)		8.94 (6.00)		9.48 (6.34)	
Number of technology supports	2.26 (1.39)		1.80 (1.00)		2.15 (1.32)	
Developmental delay		136 (85.5)		42 (85.7)		178 (85.6)
Behavioral problem		22 (13.8)		8 (16.3)		30 (14.4)
Palliative care required		49 (30.8)		14 (28.6)		63 (30.3)
Caregiver Clinical Variables						
Mental health problem		26 (16.4)		19 (38.8)		45 (21.6)
Chronic medical condition		12 (7.5)		9 (18.4)		21 (10.1)
Interpersonal violence/trauma		4 (2.5)		15 (30.6)		19 (9.1)

### Child Welfare System Involvement

Of the 208 children with medical complexity who met study inclusion criteria, 49 (23.6%) had known past or present child welfare system involvement. The primary reason for child welfare referral was neglect (n = 16; 32.7% - half of which was documented as medical neglect), intimate partner violence (n = 10; 20.4%), physical abuse (n = 6; 12.2%), emotional abuse (n = 2; 4.1%), sexual abuse (n = 1; 2.0%), other reason such as caregiver capacity and voluntary involvement with no identified protection concern (n = 9; 18.4%), or unknown (n = 5; 10.2%). Of these 49 children, investigation outcomes showed that 19 (38.8%) were known to be placed in temporary or permanent care in the past or present, including foster care, kinship care, residential care, and adoption. This represents 9.1% of the total sample.

### Sociodemographic Variables

The children in the sample ranged in age from 2 months to 17 years (M = 54.7 months; SD = 55.9 months) at the time of referral to the Complex Care Program; 103 (49.5%) were male. Relationship status for 146 (70.2%) primary caregivers was married/common-law. Total annual family income was estimated to be low for 86 (41.3%), and 155 (74.5%) received some type of financial assistance from government or charity sources for out-of-pocket healthcare-related expenses.

### Child Clinical Variables

The mean number of health diagnoses was 13.8 (SD = 4.8; range = 3–26), most commonly involving the neurological, gastrointestinal, and respiratory systems. On average, the number of hospitalizations in the current hospital since birth was 12.8 (SD = 12.0; range = 0–74), and the number of days hospitalized as an inpatient since birth was 168.0 (SD = 163.8; range = 0–1192). The mean number of prescription medications was 9.5 (SD = 6.3; range = 0–29). The mean number of medical technology supports was 2.2 (SD = 1.3; range = 0–7). Most children (n = 178; 85.6%) had a diagnosed developmental delay, and 30 (14.4%) had documented behavioral problems. Palliative care was required for 63 (30.3%).

### Caregiver Clinical Variables

For 21.6% (n = 45) of children, one or both caregivers had a documented mental health problem, most commonly anxiety, depression, or profound distress and difficulty coping with the child’s health status. For 10.1% (n = 21) of children, one or both caregivers had a documented chronic medical condition or physical limitation. For 9.1% (n = 19) of children, one or both caregivers had a documented history of interpersonal violence or trauma.

### Factors Associated With Child Welfare System Involvement

Unadjusted and adjusted models (controlling for child age and sex) examining the associations between sociodemographic, child, and caregiver characteristics and child welfare system involvement are presented in Table 2. The documented presence of caregiver mental health problems (aOR = 3.19, 95%CI = 1.55–6.56), caregiver chronic medical conditions or physical limitations (aOR = 2.86, 95%CI = 1.09–7.47), and caregiver history of interpersonal violence or trauma (aOR = 17.58, 95%CI = 5.43–56.98) were associated with increased likelihood of child welfare system involvement, after adjusting for child age and sex. Caregiver married/common-law relationship status (aOR = 0.35, 95%CI = 0.16–0.74) and, to a lesser extent, higher number of medical technology supports (aOR = 0.75, 95%CI = 0.57–0.99) were associated with lower likelihood of child welfare system involvement.

**Table 2.** Child and Caregiver Variables Associated With Child Welfare System Referral Among Children With Medical Complexity.

	Unadjusted OR (95%CI)	Adjusted OR (95%CI)^a^
Sociodemographic Variables		
Caregiver relationship status (married/common law)	0.33 (0.16–0.69)	**0.35 (0.16**–**0.74)**
Family income (low)	2.58 (0.97–6.87)	2.51 (0.92–6.82)
Financial assistance (received)	2.11 (0.46–9.78)	2.10 (0.45–9.88)
**Child Clinical Variables**		
Number of health diagnoses	1.03 (0.97–1.10)	1.03 (0.96–1.10)
Number of hospitalizations	0.99 (0.97–1.03)	0.99 (0.97–1.02)
Number of days in hospital	1.00 (0.99–1.00)	1.00 (0.99–1.00)
Number of technology supports	**0.75 (0.57**–**0.98)**	**0.75 (0.57**–**0.99)**
Developmental delay	0.98 (0.39–2.47)	1.02 (0.41–2.53)
Behavioral problem	1.26 (0.52–3.08)	1.22 (0.50–2.93)
**Caregiver Clinical Variables**		
Mental health problem	**3.24 (1.59**–**6.60)**	**3.19 (1.55**–**6.56)**
Chronic medical condition	**2.76 (1.09**–**7.00)**	**2.86 (1.09**–**7.47)**
Interpersonal violence or trauma	**17.10 (5.33**–**54.74)**	**17.58 (5.43**–**56.98)**

*Note.* OR = Odds Ratio; CI = Confidence Interval.

^a^ ORs adjust for child age at the time of referral and child sex.

^b^ Numbers in bold equal significant at *p* < .05.

## Discussion

This study examined incidence rates of, reasons and risk factors for, and investigation outcomes subsequent to child welfare system involvement in a sample of 208 children with medical complexity followed at a pediatric hospital-based complex care program in Canada. Results indicate relatively high rates of child welfare system involvement and placement in care among children with medical complexity, with caregiver mental health problems, chronic medical conditions, and interpersonal violence or trauma increasing risk; and caregiver relationship status and, to a lesser extent, medical technology dependence decreasing risk. These findings advance the state of our empirical understanding of this small and understudied subpopulation of children and draw attention to important implications for clinical practice and policy.

Notably, we found that nearly one-quarter (23.6%) of children with medical complexity had known past or present involvement with the child welfare system, most commonly for concerns of neglect. Of this subset of children referred to the child welfare system, more than one-third (38.7%) were placed in temporary or permanent out-of-home care following investigation. These rates of system involvement and placement in care are disproportionately greater than rates found in the general population of children. In Ontario in 2018, for example, there were an estimated 63 maltreatment-related investigations per 1,000 children and no resultant out-of-home placement in 97% of investigations (Fallon et al., 2020). The higher figures observed in the current study are, however, comparable to those identified among children with chronic illnesses. For instance, Azzopardi et al. (2014) reported that 43% of their sample of 134 children with HIV followed at a pediatric hospital-based infectious diseases program had documented past or present involvement with the child welfare system, most frequently for neglect, with a roughly equivalent rate of placement in care as the current study (38.6%) and similar associated risk factors. More than 90% of children referred to child protection services for medical neglect specifically have been shown to have serious chronic diseases such as diabetes (Fortin et al., 2016).

While many families demonstrate remarkable resilience in the face of extraordinary stress and childcare demands, we found generally high levels of documented caregiver adversities across economic, social, and health domains. This echoes the results of Cohn et al.’s (2020) literature review demonstrating poorer health outcomes among caregivers of children with chronic illness compared to those without. Of the mitigating variables investigated in our study, caregiver history of mental health problems, chronic medical conditions or physical limitations, and interpersonal violence or trauma were shown to be significantly associated with increased likelihood of child welfare system involvement, as hypothesized. These findings are consistent with other research investigating caregiver risk factors, including child welfare incidence data showing that a considerable proportion of primary caregivers in substantiated child maltreatment investigations have mental and physical health issues, as well as their own experiences with violence (Fallon et al., 2020; Public Health Agency of Canada, 2010). Such hardships may intensify parenting stress and impede coping ability.

Caregiver relationship status of married/common-law (vs. single) was associated with decreased likelihood of child welfare system involvement in our study, possibly suggesting that the benefits of family integration, such as emotional support, caregiving respite, and financial security, may operate as a protective factor. Children who were dependent on a larger number of medical technologies for basic biological functions, such as ventilators and feeding tubes, were also somewhat less likely to have involvement with the child welfare system. While merely speculative, this unexpected inverse relationship may be explained by the supportive presence of more healthcare providers in the lives of these families or increased access to financial and respite resources as a result of being more medically fragile. Alternately, the children requiring the most interventions may be the least likely to understand and disclose maltreatment due to more severe functional impairment, and therefore the least likely to come to the attention of authorities. Closer examination of these compensatory conditions are notable areas for future research.

Inconsistent with our hypotheses, most of the clinical features related to the child’s health status (number of health diagnoses, hospitalizations, days in hospital, development delay, behavioral problem) were not significantly associated with likelihood of child welfare system involvement. The medical profiles of all children included in this sample were highly complex and thus, it is plausible that there may have been little meaningful difference in care demands and related impacts for families along the health spectrum. Also unexpectedly, low family income and financial assistance were not predictive of higher odds of child welfare system involvement. In contrast, prior research has linked socioeconomic adversity with medical complexity status in children (Yu et al., 2021), as well as with child maltreatment and protection investigation incidence more generally (Fallon & Van Wert, 2017). Material resource-related risk posed to children with medical complexity is an important avenue for further study.

When children have complicated and chronic medical diagnoses, there is a natural tendency for families and healthcare providers to focus their limited time, energy, attention, and resources on meeting the care needs of the child. The care needs of the caregiver often get overlooked in the process. As Zuckerman (2016) and Rotberg et al. (2020) emphasize, however, children are best helped by helping their parents. The main findings of our study point to the need for multilevel support services for caregivers of children with medical complexity in pediatric healthcare settings to decrease caregiving burden, financial strain, and emotional distress; and to increase caregiving capacity, coping skills, and social support. A range of family-level and system-level interventions have been shown to alleviate caregiver stress, particularly those which focus on streamlining services and reducing caregiving pressures (Edelstein et al., 2016). Mobilizing this support demands an interprofessional approach to holistic medical and psychosocial care wherein pediatricians and allied health practitioners assume vital complementary roles.

Effective clinical assessment and intervention efforts targeting caregivers have the potential to mitigate risk for child maltreatment exposure and child welfare system involvement. This pathway of care could be set in motion with a trusting relationship and routine screening using brief validated instruments on admission for caregiver physical and mental health status, parenting stress and social support, exposure to violence and trauma, and other domains of risk (e.g., Parenting Stress Index, Abidin, 2012; Trauma Screening Questionnaire, Brewin et al., 2002; Psychosocial Assessment Tool, Kazak, 2006; Patient Health Questionnaire, Kroenke et al., 2009). This would help to normalize and diminish common feelings of shame and stigma associated with the struggles of caring for a child with medical complexity; and it would systematically identify caregivers requiring more in-depth clinical assessment and treatment, ultimately bolstering support and reducing risk. Most pediatric healthcare providers can feasibly deliver simple strengths-based interventions, such as psychoeducation and problem-solving skills training, to caregivers of children with complex medical histories in busy pediatric settings (Rotberg et al., 2020). For those with greater support needs, collaboration with primary care providers or referrals to community-based resources may be necessary for more intensive evidence-informed services. In their review of randomized control trials evaluating psychological interventions designed for parents of children with chronic illness, Eccleston et al. (2015) found that, while there was little evidence to support their efficacy overall, some therapies, such as cognitive-behavioral therapy and problem-solving therapy, were shown to reduce negative outcomes in parents and children in the short- and long-terms. Home visiting programs have also demonstrated promising evidence for improving maternal and child health and reducing child maltreatment (Avellar & Supplee, 2013; Jack et al., 2015). To foster trust and rapport, tailored interventions should be nonjudgmental, compassionate, and culturally sensitive as social constructions of illness and child-rearing norms vary across diverse cultural backgrounds (Garwick et al., 1998; Kuo et al., 2016).

Though comprising a small fragment of the pediatric population, our findings suggest that children with medical complexity deemed at risk of harm are subject to exceedingly high child welfare resource utilization, including specialized medical foster homes and residential facilities. Placement in care can have unintended traumatic effects, particularly when placement breakdowns beget multiple transitions in care, as is commonly the case for children with special needs (Szilagyi et al., 2015). In their interactions with the child welfare system, pediatric healthcare providers are well-positioned to raise awareness, educate, and advocate in the best interests of the chronically ill child and family. It is important to note that not all child welfare referrals in this study were mandated in response to imminent threats to safety. Some caregivers sought out child welfare support and resorted to relinquishing custody in order to obtain essential services when childcare demands overwhelmed their caregiving capacity at home, signifying deficiencies in alternative, less intrusive systems of support (Ombudsman Ontario, 2005). This underscores the significance of public policies that serve to avert reliance on the child welfare system as a proxy for equitable, accessible, and responsive health and social services. This calls for a policy framework prioritizing the social determinants of health (Mikkonen & Raphael, 2010), with commitment to sustainable funding for complex care programs integrating pediatric medical care and adult mental healthcare, enhanced homecare support, caregiving respite resources, and guaranteed financial coverage for essential healthcare expenditures. Taken together, the proposed clinical strategies and policy directions promote optimal caregiver health and well-being, and by extension, optimal child development and safety.

## Limitations

The findings of this study should be interpreted within the parameters of its methodological limitations. Foremost, conclusions of cause-and-effect relationships and generalizability of results beyond this sample are precluded by study design and should therefore be addressed in future lines of pediatric health and maltreatment research. Although our sample size was adequate to detect small to medium effects, some odds ratios had large confidence intervals, suggesting less precision in our estimates. Further research replicating the results found herein are necessary for drawing firm conclusions on risk and protective factors associated with child welfare system involvement among children with medical complexity.

In keeping with the general shortcomings of cross-sectional, single-site chart reviews (Gearing et al., 2006), prospective variable construction and comparative analyses with control groups were unfeasible. As is typical in retrospective chart review methodology, the information in the data files were contingent on the contents of existing health records, with the original purpose of clinical documentation. The data included in our review were collected at one point in time at one setting and therefore may not be representative of populations across different sites or evolving trends. Moreover, we did not have definitive data on the proportion of children with chronic conditions that resulted from maltreatment, so we could not examine chronology of child welfare system involvement (though we expect this would affect less than 2% of the sample based on our clinical experience). Additionally, several important sociodemographic characteristics, including race/ethnicity and caregiver age, were not consistently charted in the health record and consequently could not be considered in the analyses. This represents a critical direction for future research given known disparities in healthcare and child welfare systems.

Rates of child welfare system involvement, placement in care, and all caregiver risk factors were dichotomized on the basis of their *known* presence according to collateral or caregiver report. Universal screening with standardized assessment measures was not the usual standard of care. The absence of documentation does not inherently equate to the absence of incidence or risk. It has been well established that adverse childhood experiences, including child maltreatment, are substantially under-reported and under-documented in electronic health records (Karatekin et al., 2017). Recorded rates in this study are thus likely to be an under-representation of true scope. This suggests the need for future research to systematically track hospital-based and child welfare system-based data on maltreatment of children with medical complexity. Further empirical consideration should also be given to the chronology of child welfare referral and risk conditions, the cumulative effects of independent and overlapping risk factors, and the differential impacts of various medical diagnoses subgroups.

## Conclusion

This study highlighted the critical interface of the child welfare system with the healthcare of children with medical complexity. Since these children are inherently defined by intense care needs necessitating extensive support across providers, institutions, and time, the findings are, to some degree, not unexpected. Families caring for chronically ill children at home undergo exceptionally high caregiving responsibility and stress. Responsive public policies and clinical intervention strategies are required to ensure that caregivers can be better supported, families can be strengthened, and child maltreatment and its traumatic impacts can be prevented. Stronger partnerships and enhanced collaboration among pediatric and adult health, mental health, and child welfare sectors are one avenue through which children can be more effectively protected.
